# FGR Diagnosis with EFW <10% versus AC <10%: Differences in Clinical Presentation, Pregnancy Outcomes, and Correlation with Placental Lesions of Malperfusion

**DOI:** 10.1055/a-2573-4517

**Published:** 2025-04-30

**Authors:** Megan Savage, Luiza Perez, Natalie Nguyen, Stephen Chasen

**Affiliations:** 1 Division of Maternal Fetal Medicine, Department of Obstetrics and Gynecology, Lenox Hill Hospital, Zucker School of Medicine at Hofstra/Northwell, New York, New York; 2 Department of Obstetrics and Gynecology, Weill Cornell Medical College, New York, New York; 3 Division of Maternal Fetal Medicine, Department of Obstetrics and Gynecology, Weill Cornell Medical College, New York, New York

**Keywords:** fetal growth restriction, placental pathology, malperfusion, obstetrical outcomes

## Abstract

**Objective:**

This study aimed to identify what biometry is most predictive of placental malperfusion and obstetrical outcomes.

**Study Design:**

Retrospective cohort study comparing pregnancies diagnosed with fetal growth restriction (FGR) from 2018 to 2020. Pregnancies with estimated fetal weight (EFW) < 10th percentile were characterized as the “EFW” group, and those with normal EFW but abdominal circumference (AC) < 10th percentile were characterized as the “AC” group. Mann–Whitney U, Fisher's exact test, and chi-square were used for statistical comparison.

**Results:**

A total of 318 pregnancies were included, with 250 and 68 in EFW and AC groups, respectively. There were no significant differences in demographics between groups. The diagnosis was earlier in the EFW group (33 [30–36] vs. 35 [32–36] weeks; *p* = 0.001), with a higher proportion diagnosed at < 32 weeks. Delivery was also earlier in the EFW group (37 [35–38] vs. 38 [36–39] weeks; *p* = 0.01), with a higher rate of delivery <34 weeks compared with the AC group. Diagnosis at < 32 weeks was associated with higher rates of maternal (75.5 vs. 51.4%; *p* < 0.001) and fetal (25.5 vs. 14.6%; *p* = 0.02) malperfusion. After initial diagnosis, follow-up ultrasound was not consistent with FGR in 11.0% of cases, and this was more common in the AC group (19.1 vs. 8.7%; *p* = 0.03). “Resolution” of FGR was associated with lower rates of maternal malperfusion compared with persistent findings of FGR (28.5 vs. 63.3%; *p* < 0.001).

**Conclusion:**

In the cohort with FGR based on EFW <10th percentile, diagnosis and delivery were earlier. There was also a higher rate of delivery <34 weeks in the EFW group. There were no significant differences in the rate of placental lesions of maternal or fetal malperfusion based on diagnostic criteria of FGR however a diagnosis <32 weeks was associated with higher rates of malperfusion. Diagnosis based on small AC was more likely to “resolve” on follow-up and this was associated with lower rates of maternal malperfusion.

**Key Points:**

- FGR based on EFW <10th percentile, diagnosis and delivery were earlier.
- FGR diagnosed <32 weeks is associated with higher rates of malperfusion.
- FGR diagnosis based on small AC was more likely to “resolve” on follow-up.

Intrauterine fetal growth restriction (FGR) is diagnosed in approximately 10% of pregnancies. 1 However, the true prevalence of FGR is difficult to ascertain. It can be difficult to delineate if the fetal weight is due to nonpathological constitutional growth or a consequence of maternal, fetal, or placental pathologies. In addition, the diagnosis of FGR can be confounded by evolving diagnostic criteria.

The most recent Society for Maternal–Fetal Medicine (SMFM) guidelines define FGR as an estimated fetal weight (EFW) or abdominal circumference (AC) that is less than the 10th percentile for gestational age. 2 This new definition of growth restriction is more conservative and results in more pregnancies receiving a diagnosis of intrauterine growth restriction. This is consequential as intrauterine growth restriction can be associated with adverse perinatal outcomes and requires the patient to present for additional antenatal testing. 3 This can include visits with the healthcare team one to three times per week from diagnosis until delivery. This testing, although indicated, can be cumbersome for the patient.

Identifying what types of biometry are most predictive of placental malperfusion and poor obstetrical outcomes would aid in patient counseling and help clinicians optimize the frequency of antepartum surveillance and timing of delivery. Our objective was to assess if the amended definition of intrauterine growth restriction is associated with clinically significant rates of obstetrical outcomes. In addition, we aimed to assess if the new definition of intrauterine growth restriction is associated with placental histological findings such as lesions of malperfusion.

## Materials and Methods

An ultrasound database was queried to identify patients with the diagnosis of intrauterine growth restriction. This was a retrospective cohort study comparing pregnancies diagnosed with FGR from 2018 to 2020. Data was collected on the type of intrauterine growth restriction, sonographic parameters for this diagnosis, patient demographics, medical history (to include risk factors for intrauterine growth restriction), placental pathology, and obstetrical outcomes.

The study population included pregnant females over the age of 18 who presented to a tertiary-care academic institution ultrasound suite with a diagnosis of intrauterine growth restriction. Pregnancies with fetal anomalies and genetic conditions were excluded. Pregnancies with EFW <10th percentile were characterized as the “EFW” group, and those with normal EFW but AC < 10th percentile were characterized as the “AC” group. All pregnancies underwent surveillance consistent with SMFM recommendations. The primary outcome was to determine if there was a difference in obstetrical outcomes (age of diagnosis of FGR, gestational age at delivery, AGPAR scores, intrauterine fetal demise) between the two groups. The secondary outcome was to determine if there was a difference in lesions of maternal or fetal malperfusion. Pregnancy characteristics and outcomes were compared with a Mann–Whitney U, Fisher's exact test, and chi-square statistical comparison. Continuous data are expressed as median (interquartile range). Placental pathology was performed by perinatal pathologists who identified lesions of maternal and fetal malperfusion. The placental lesions of maternal and fetal malperfusion are outlined in Table 1 . The frequency of lesions was compared in the cohorts. Mann–Whitney *U* and Fisher's exact test were used for statistical comparison.

**Table 1:** The placental lesions of maternal and fetal malperfusion were identified by perinatal pathologists

Maternal lesions of malperfusion	Fetal lesions of malperfusion
• Villous infarct • Increased perivillous fibrin deposition • Accelerated villous maturity • Increased syncytial knots • Multifocal villous agglutination • Distal villous hypoplasia • Decidual vasculopathy • Intraplacental hematoma • Multiple chorionic cysts	• Intramural fibrin deposition • Avascular villi • Thrombosis of fetal vessels • Villous stromal vascular karyorrhexis • Stem vessel obliteration

## Results

A total of 318 pregnancies were included, with 250 and 68 in the EFW and AC groups, respectively. All but two cases in the EFW group also had AC < 10th percentile. Median maternal age was 34 (31–37) with FGR diagnosed at 33 (30–36) weeks. There were no significant differences in age, ethnicity, BMI, or medical co-morbidities between groups. Rates of oligohydramnios and Doppler abnormalities were similar. The diagnosis was earlier in the EFW group (33 [30–36] vs. 35 [32–36] weeks; *p* = 0.001), with a higher proportion diagnosed at <32 weeks ( Table 2 ).

**Table 2:** Pregnancy outcomes for FGR with diagnosis due to EW < 10th percentile and AC < 10th percentile

	EFW < 10th percentile ( *n* = 250)	AC < 10th percentile ( *n* = 68)	*p* -Value
Gestational age at diagnosis (wk)	33 (30–36)	35 (32–36)	0.001
Diagnosis <32 wk	36.8%	20.6%	0.01
Gestational age at delivery (wk)	37 (35–38)	38 (36–39)	0.01
Delivery at <34 wk	20.4%	8.8%	0.03
Birthweight (g)	2,250 (1,820–2,581)	2,450 (2,288–2,688)	<0.001
Oligohydramnios at diagnosis	3.6%	1.5%	0.70
UA Doppler abnormality at diagnosis	27.6%	25.0%	0.76
“Resolution” of FGR on follow up ultrasound	8.7%	19.1%	0.03

Abbreviations: AC, abdominal circumference; EFW, estimated fetal weight; FGR, fetal growth restriction; UA, umbilical artery.

The median gestational age at delivery was 37 weeks. Delivery was earlier in the EFW group, 37 (35–38) versus 38 (36–39) weeks ( *p* = 0.01), with a higher rate of delivery < 34 weeks compared with the AC group ( Table 2 ). The trimmed placental weight was greater in the AC group, 340 (288–410) versus 319 (268–377) g; ( *p* = 0.02).

Lesions of maternal malperfusion were seen in 59.4% of pregnancies, with multiple lesions in 37.1%. Lesions of fetal malperfusion were seen in 18.2%, with multiple lesions in 8.2% ( Table 3 ). Diagnosis at < 32 weeks was associated with higher rates of maternal (75.5 vs. 51.4%; *p* < 0.001) and fetal (25.5 vs. 14.6%; *p* = 0.02) malperfusion. There were no differences in the rate of lesions of maternal or fetal malperfusion between the groups.

**Table 3:** Lesions of maternal and fetal malperfusion per cohort

	EFW <10th percentile ( *n* = 250)	AC <10th percentile ( *n* = 68)	*p* -Value
Placental weight (g)	319 (268–377)	340 (288–410)	0.01
Placental lesions of maternal malperfusion	61.6%	51.5%	0.16
Multiple lesions of maternal malperfusion	38.8%	30.9%	0.26
Placental lesions of fetal malperfusion	18.4%	17.6%	1.0
Multiple lesions of fetal malperfusion	7.6%	10.3%	0.46

Abbreviations: AC, abdominal circumference; EFW, estimated fetal weight.

After initial diagnosis, follow-up ultrasound was not consistent with FGR in 11.0% of cases, and this was more common in the AC group (19.1 vs. 8.7%; *p* = 0.03; Table 2 ). “Resolution” of FGR was associated with lower rates of maternal malperfusion compared with persistent findings of FGR (28.5 vs. 63.3%; *p* < 0.001). There was no difference in the rate of low Apgar scores, and there were no stillbirths in either group.

## Discussion

FGR can occur in 10% of pregnancies and the most common cause, suboptimal placental perfusion, is attributed to 25 to 30% of these cases. 4 FGR is a leading cause of infant morbidity and mortality and antepartum diagnosis allows for increased antepartum surveillance and medically indicated early-term or preterm delivery. 5

In a prospective study of 1,000 low-risk pregnancies, an AC of less than the 10th percentile was found to have diagnostic accuracy similar to EFW less than the 10th percentile for the prediction of SGA. 6 Expanding the diagnosis of FGR to include an AC less than the 10th percentile aims to increase the frequency of antepartum diagnosis and pregnancy intervention to reduce adverse perinatal outcomes.

However, it has been estimated that approximately 18 to 22% of fetuses diagnosed with FGR will be constitutionally small at birth. 7 As a result, many fetuses diagnosed with FGR are not always SGA at birth and these patients may have undergone unnecessary antenatal testing and subsequent early delivery. Our data suggests that many of these pregnancies with a diagnosis of FGR will not have lesions of maternal or fetal malperfusion, abnormal umbilical artery dopplers, or poor birth outcomes.

There were some notable differences between the two cohorts. In the cohort with FGR based on EFW <10th percentile, diagnosis, and delivery were earlier compared with the cohort with AC <10th percentile. There was also a higher rate of delivery <34 weeks in the EFW group. There were no significant differences in the rate of placental lesions of maternal or fetal malperfusion based on diagnostic criteria of FGR however a diagnosis <32 weeks was associated with more rates of malperfusion.

Diagnosis based on small AC was more likely to “resolve” on follow-up compared with diagnosis based on EFW <10th percentile and this was associated with lower rates of maternal malperfusion. Our data suggest that using AC alone to diagnose FGR in those with EFW > 10th percentile could identify a milder form of FGR, though large studies are necessary to determine if the expanded diagnosis is clinically beneficial.

Strengths of this study include a dedicated placental pathologist and a patient population that adheres to recommended surveillance timelines. The data are limited as we were unable to track long-term neonatal outcomes. This study was also performed at a single clinical site and protocols for the diagnosis of FGR and antenatal testing can differ across obstetrical ultrasound units. Additional research is needed to identify antenatal diagnostic criteria that are associated with placental malperfusion. Identifying these pregnancies with FGR and suspected suboptimal placental perfusion would help clinicians decisions regarding the frequency of antenatal testing and the timing of delivery.
